# Effects of the Fungal Endophyte *Epichloë festucae* var. *lolii* on Growth and Physiological Responses of Perennial Ryegrass cv. Fairway to Combined Drought and Pathogen Stresses

**DOI:** 10.3390/microorganisms8121917

**Published:** 2020-12-02

**Authors:** Fang Li, Tingyu Duan, Yanzhong Li

**Affiliations:** 1 Key Laboratory of Grassland Livestock Industry Innovation, Ministry of Agriculture and Rural Affairs, Lanzhou University, Lanzhou 730020, China; lif2013@lzu.edu.cn (F.L.); liyzh@lzu.edu.cn (Y.L.); 2 State Key Laboratory of Grassland Agro-Ecosystems, Lanzhou University, Lanzhou 730020, China; 3 Engineering Research Center of Grassland Industry, Ministry of Education, Lanzhou University, Lanzhou 730020, China; 4 College of Pastoral Agriculture Science and Technology, Lanzhou University, Lanzhou 730020, China

**Keywords:** fungal endophyte, pathogen, soil water regime, plants growth, physiological responses

## Abstract

Perennial ryegrass (*Lolium perenne*) is widely cultivated around the world for turf and forage. However, the plant is highly susceptible to disease and is sensitive to drought. The present study aims to determine the effect of the fungal endophyte *Epichloë festucae* var. *lolii* of perennial ryegrass on the combined stresses of drought and disease caused by *Bipolaris sorokiniana* in the greenhouse. In the experiment, plants infected (E+) or not infected (E−) with the fungal endophyte were inoculated with *Bipolaris sorokiniana* and put under different soil water regimes (30%, 50%, and 70%). The control treatment consisted of E+ and E− plants not inoculated with *B. sorokiniana*. Plant growth, phosphorus (P) uptake, photosynthetic parameters, and other physiological indices were evaluated two weeks after pathogen infection. The fungal endophyte in E+ plants increased P uptake, plant growth, and photosynthetic parameters but decreased the malondialdehyde concentration, proline content, and disease incidence of perennial ryegrass (*p* < 0.05). E+ plants had the lowest disease incidence at 70% soil water (*p* < 0.05). The study demonstrates that the fungal endophyte *E. festucae* var. *lolii* is beneficial for plant growth and stress tolerance in perennial ryegrass exposed to the combined stresses of drought and *B. sorokiniana*.

## 1. Introduction

Plants are often exposed to various abiotic and biotic stresses such as drought, temperature extremes, salinity, heavy metals, and disease. Usually, plants encounter these adverse conditions simultaneously, thereby facing combined stresses such as drought and disease. These stresses may disrupt cellular homeostasis, and the coupling of stresses induces numerous changes that manifest as decreased plant growth and productivity [1]. Subsequently, virtually every aspect of plant physiology and biochemistry is affected, including photosynthetic processes, nutrient uptake, enzyme activity, and osmotic adjustment [1]. 

Perennial ryegrass (*Lolium perenne*) is an important turfgrass and forage that is widely cultivated around the world [2]. However, this forage crop is highly susceptible to diseases [3] and sensitive to soil moisture deficit (drought) [4]. Hence, disease and drought significantly limit the use of perennial ryegrass in China and other parts of the world [5]. Leaf spot disease caused by *Bipolaris sorokiniana* is a serious disease that causes various symptoms, ranging from necrotic lesions on the leaf tissue to plant blight or death [6]. Tian et al. [7] reported that *B. sorokiniana* could cause disease symptoms in attached and detached perennial ryegrass leaves and in field conditions.

Fungal endophytes (*Epichloë*) are ubiquitous microbes associated with most Poaceae plant species [8]. These fungi are exclusively seed-borne, mostly spread via vertical transmission [9], and are located in the intercellular spaces of aboveground vegetative tissues [10]. The symbiotic relationship between fungal endophytes and grasses is mutualistic [11]; the fungal endophytes gain shelter, nutrition, and dissemination via host plants [12,13]. Meanwhile, the fungal endophytes enhance plant growth [14] and nutrient uptake [15] in host plants. Of note, this symbiotic relationship can increase plant resistance to abiotic [16,17] and biotic stresses [18,19].

The symbiosis of perennial ryegrass and *Epichloë festucae* var. *lolii* is prevalent in grassland agricultural ecosystems. The grass–endophyte association increases the plant’s stress resistance by augmenting the following defense mechanisms: enhanced nutrient status and plant growth [15]; production of alkaloid toxins and protection of host grasses from grazing animals [8]; activation of antioxidant enzyme activities such as catalase (CAT), peroxidase (POD), and polyphenol oxidase (PPO) to suppress reactive oxygen species (ROS) production [7,19,20]; production of antifungal substances to inhibit pathogen growth [21]; induction of osmotic adjustment [22]. Nevertheless, the effect of a symbiont on stresses depends on the plant genotype [23], fungal endophyte strain [24], and the environmental conditions [24].

The individual effects of drought and disease stresses on plants have been widely studied [19,25,26,27]. Our previous study showed that when perennial ryegrass is exposed to leaf spot caused by *B. sorokiniana*, the coinfection with arbuscular mycorrhizal (AM) fungus and grass endophyte positively impacts antioxidant enzymes activities, malonaldehyde (MDA) contents, and hydrogen peroxide (H_2_O_2_) concentrations [19]. In Li et al. [25], we tested the growth and physiological responses of perennial ryegrass to an AM fungus and an *Epichloë* endophyte under different soil water contents; the study showed that the plant–AM–endophyte symbiosis alleviates the damage caused by drought stress by promoting P uptake, photosynthesis, and the accumulation of osmoregulatory substances. Another study by our research group [26] tested the individual effect of AM fungus on disease and drought stress resistance in perennial ryegrass; the results revealed that the AM fungus could improve P uptake and growth of perennial ryegrass to alleviate the damage caused by biotic and abiotic stresses. Xia et al. [27] examined the effect of the endophyte *Epichloë gansuensis* on the powdery mildew disease caused by *Blumeria graminis* and the growth of the host grass *Achnatherum inebrians* under four soil water conditions. Their findings demonstrated that the presence of the *Epichloë* endophyte reduces the ability of *B. graminis* to colonize *A. inebrians* and enhances host plant growth at all tested soil water conditions.

The fungal endophyte *E. festucae* var. *lolii* may provoke a similar response in perennial ryegrass infected by *B. sorokiniana*. Therefore, a greenhouse experiment was established to study the effects of fungal endophyte on the combination of drought and disease stresses. In the study, perennial ryegrass with or without fungal endophytes and infected or uninfected by the pathogen were put under different soil water regimes. Plant growth, phosphorus (P) uptake, photosynthetic parameters, enzyme activities, and osmotic adjustment substances were subsequently measured.

## 2. Materials and Methods

### 2.1. Plants, Fungi, and Potting Medium

*Bipolaris sorokiniana* was isolated from diseased leaves of perennial ryegrass and confirmed by Koch’s postulates to be the causative pathogen of leaf spot disease. The symptomatic leaves were surface-sterilised with 75% alcohol and 1% sodium hypochlorite (NaClO) for 30 s each. The sterilized leaves were then cut into small pieces, placed onto potato dextrose agar (PDA), and transferred to a 25 °C incubator. Four days later, the isolates were subcultured onto fresh PDA media and maintained for four weeks at 25 °C. To prepare the inoculum, 10 mL sterilized water was added to each Petri dish, and the fungal colonies were scraped with a sterile spreader. A conidial suspension was produced by filtering the fungus through four layers of sterilized cheesecloth, and the final concentration was determined and adjusted to approximately 1 × 10^6^ conidia mL^−1^ using a hemocytometer.

Perennial ryegrass (“Fairway” cultivar) seeds infected (E+) or uninfected (E–) by the systemic endophyte *Epichloë festucae* var. *lolii* were collected from plants with a previously verified *Epichloë* infection status, checked using the method of Nan [28]. The seeds were stored at a constant temperature (4 °C) at the Forage and Turfgrass Seed Quality Supervision and Testing Center (Lanzhou), China, and replanted each year to collect seeds with and without the fungal endophyte. Before the experiment, 50 seed samples, each from E+ and E– plants, were randomly selected and the aleurone layer microscopically examined for *Epichloë* hyphae by staining with an aniline blue solution. The endophyte infection rate of E+ seeds was >98%, while the endophyte−free infection rate of E– seeds was >99%.

The potting medium used in this study consisted of a soil and sand mixture (1:1) sieved through a 2-mm sieve and autoclaved twice at 121 °C for 1 h at a 3-day interval between sterilizations. The potting mixture had a pH of 6.58 and a plant-available P-level of 15.87 mg kg^−1^. The water holding capacity of the potting medium was measured using the weighing method, as described by Yu et al. [29]. The pots were 6 × 22 × 15 cm in size, and each contained 1.5 kg of the potting mix.

### 2.2. Experiment Design and Management

A full factorial design with three−factor experiments was adopted: fungal endophyte (two levels) × pathogen (two levels) × soil water regime (three levels) = 12 treatments, each with four replicate pots.

Seeds of E+ and E– perennial ryegrass were surface-sterilized with 75% ethanol and 1% NaClO each for 60 s. The sterilized seeds were then placed in a 25 °C; incubator to germinate. After 5 d germination, six germinated E+ or E– seeds were transplanted into the same pot. Plants were grown in a glasshouse maintained at a 12 h photoperiod, a relative humidity range of 65–90%, and temperatures of 23–28/20–25 °C (day/night). The plants were watered with a modified Long Ashton nutrient solution [30] every other day.

Two leaf-sheaths of each seedling were checked for the infection of hyphae of *E. festucae* var. *lolii* via 0.2% aniline blue staining and observed at 40× magnification [31] at four weeks. The plants were not watered from then on to reduce the soil water content of each pot to 30% of water-holding capacity. Then, three different water treatments were established by weighing each pot, including 30%, 50%, and 70% soil water regimes for E+ and E− treatments, respectively. Each pot was weighed every day and water was added to maintain soil moisture content at 30%, 50%, or 70% soil water, respectively, and plants were grown for another four weeks, during which the shoot height and tiller numbers of each plant were determined at weekly intervals. After four weeks of water stress, 10 mL of the *B. sorokiniana* spore suspension was prepared as described above and sprayed into each pot (B+). The control (noninoculated, B−) plants were treated similarly but with sterile water. All pots were then covered with a black plastic bag for the following 48 h. Two weeks after inoculation with *B. sorokiniana*, leaf samples from each pot were collected; samples from the three plants in each pot were mixed before conducting the various tests.

### 2.3. Protocols for Measurement and Determination of the Various Parameters

#### 2.3.1. Disease Incidence

Two weeks after inoculation with *B. sorokiniana*, the plants were visually assessed for necrotic lesions, and the pathogen was reisolated. Pathogen identity was confirmed based on colony and spore morphology and sequence analysis. Then, the disease incidence was measured according to our previous study [19]. Eight or nine leaves from each of the three plants were selected, resulting in a total of 25 leaves from the three plants in each pot. Disease incidence was calculated by expressing the number of leaves with lesions as a percentage of the total number of leaves sampled from each pot (25). 

#### 2.3.2. Chlorophyll Content

Chlorophyll content was determined using a protocol modified from the method of Starnes and Hadley [32]. Approximately 0.1 g fresh leaf sample was cut into 2–3 mm length, put in 10 mL of 80% acetone, and homogenized to extract the chlorophyll. The homogenates were kept in darkness to minimize chlorophyll breakdown. Chlorophyll extracts were quantified colorimetrically with a spectrophotometer (Shimadzu, Australia) at 663 and 645 nm, using 80% acetone as a reference.

#### 2.3.3. Photosynthetic Parameters

Before harvest, three leaves with similar height and size were selected from each pot for evaluation of the photosynthetic parameters. The parameters, including the net photosynthetic rate (Pn), stomatal conductance (Gs), and transpiration rate (Tr), were determined from 9:00 to 11:00 a.m. using a Li-6400 portable photosynthesis measurement tool (LI-COR Inc., Lincoln, NE, USA). The chamber (measure window = 2 × 3 cm) was equipped with a red/blue LED light source, with the photosynthetically active radiation (PAR) set at 1200 mol m^−2^ s^−1^, the detesting conditions at T = 28 ± 1 °C, and air carbon dioxide concentration = 410 ± 10 μmol CO_2_ mol^−1^.

#### 2.3.4. Catalase (CAT) and Peroxidase (POD) Enzyme Activities

To measure antioxidant enzyme activities, ~0.1 g of fresh leaf sample from each pot was ground in sodium phosphate buffer containing polyvinyl pyrrolidine (1%). The mixture was centrifuged at 10,000 rpm at 4 °C for 15 min. CAT activity was determined at 560 nm, as described by Beers and Sizer [33], while POD activity was determined at 470 nm by the method of Chance and Maehly [34].

#### 2.3.5. Malondialdehyde (MDA)

MDA content was determined using the method of Li et al. [34]. Approximately 0.1 g fresh leaf samples from each pot were homogenized in 5 mL of 5% (*w*/*v*) trichloroacetic acid (TCA) and centrifuged at 3000 rpm at 4 °C for 10 min. Then, 2 mL of the supernatant was mixed with 2 mL of 0.67% thiobarbituric acid solution and incubated in a water bath at 100 °C for 30 min. Subsequently, the homogenates were centrifuged at 3000 rpm at 4 °C for 10 min to remove the precipitate. The absorbance was read at 600, 450, and 532 nm using a spectrophotometer.

#### 2.3.6. Proline

Proline was determined using a method modified from that of Li et al. [35]. About 0.1 g of fresh leaf samples from each treatment was homogenized in 5 mL of 3% (*w*/*v*) aqueous sulphosalicylic acid and kept in boiling water for 10 min. Then, 2 mL filtrate, 2 mL acid ninhydrin, and 2 mL glacial acetic acid were added in a vial and kept in boiling water for 30 min. The mixture was extracted with toluene, and the supernate was centrifuged at 3000 rpm for 5 min. The absorbance of the supernatant was determined at 520 nm using a spectrophotometer.

#### 2.3.7. Plant Dry Weight and Phosphorus Content

All the plant tissues remaining after the above analyses were harvested by cutting off the shoot parts at ground level, and the roots of each plant were dug up and washed. The tissues were then oven-dried at 105 °C for 20 min and at 80 °C for 48 h to constant weight to determine shoot dry weight and root dry weight. The total aboveground or underground dry weight of each plant was calculated from the ratio of fresh weight to dry weight. The dried samples were then ground by a grinding mill, and ~0.1 g of each plant powder was used to determine phosphorus concentrations using the phosphovanado–molybdate method [36]. The powder was homogenized in 5 mL of concentrated nitric acid, and the absorbance of the homogenate was evaluated by a spectrophotometer at 390 nm.

### 2.4. Statistical Analysis

The data presented in the figures are mean ± standard error of the mean for four replicates of each treatment. Data analyses were performed by SPSS 19.0 (SPSS Inc., Chicago, IL, USA) statistical analysis software. The homogeneity of variance was evaluated by Levene’s test, and a three-factor analysis of variance (ANOVA) was employed to determine the influence of the fungal endophytes on the combined stresses of drought and disease. Comparisons between means were made using Tukey’s honestly significant difference (HSD) at the *p* ≤ 0.05 level. The correlations among treatments, plant growth, P uptake, and physiological indices of perennial ryegrass were analyzed with the Pearson correlation test.

## 3. Results

### 3.1. Disease Incidence

**Figure 1:**
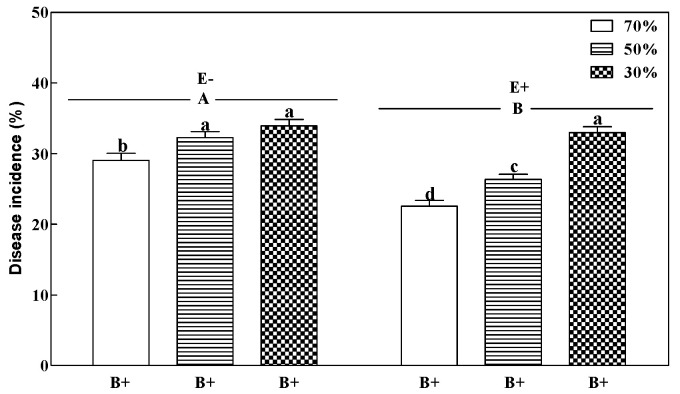
Disease incidence of perennial ryegrass infected with (E+) or without (E−) fungal endophyte at 70%, 50%, and 30% soil water regimes at harvest. Mean ± SEM of four replicates are shown. Bars topped by the same lowercase letter indicate no significant difference between soil water regimes and pathogen within fungal endophyte treatments at *p* ≤ 0.05 by Tukey’s honestly significant difference (HSD) test. Bars topped by the same uppercase letter indicate no significant difference associated with the fungal endophyte at *p* ≤ 0.05 by Tukey’s HSD test.

Plants inoculated with *B. sorokiniana* (B+) showed typical leaf spot symptoms two weeks after inoculation, while such symptoms were not found on noninoculated (B−) plants. Fungal endophyte, pathogen, and soil water regime demonstrated a 3-way interaction for disease incidence of perennial ryegrass (Table 1). The disease incidence of E+ plants demonstrated a significant increase with decreasing soil water regime (*p* < 0.05; Figure 1). However, the grass fungal endophyte (E+) significantly decreased the disease incidence by 22.4% and 18.4% at 70% and 50% soil water (*p* < 0.05) compared to treatments without the fungal endophyte (E−). The E+ plants showed the lowest disease incidence at 70% soil water (Figure 1).

### 3.2. Photosynthetic Parameters

Each of the two-way interactions for the four photosynthetic parameters was significant, except grass fungal endophyte × pathogen for the net photosynthetic rate (Pn) interaction (Table 1). The 3-way interaction was also highly significant for Pn and transpiration rate (Trans; Table 1).

**Figure 2:**
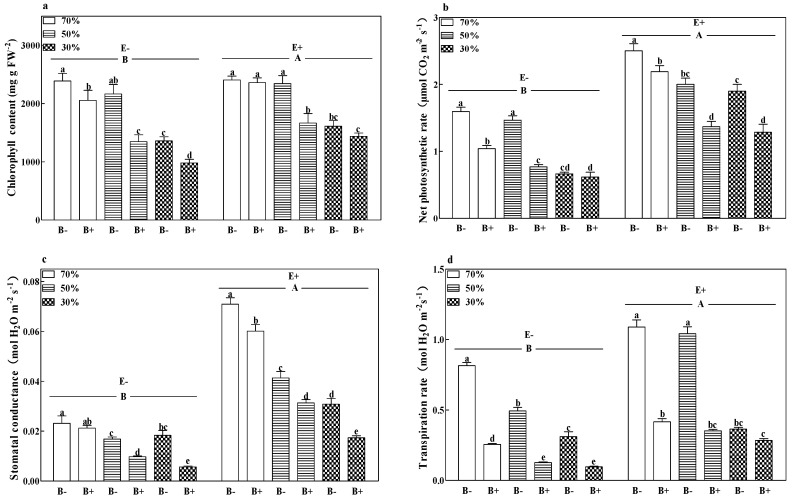
Chlorophyll content (**a**), net photosynthetic rate (**b**), stomatal conductance (**c**), and transpiration rate (**d**) of perennial ryegrass infected with (E+) or without (E−) fungal endophyte and inoculated (B+) or noninoculated (B−) with pathogen at 70%, 50%, and 30% soil water regimes at harvest. Mean ± SEM of four replicates are shown. Bars topped by the same lowercase letter indicate no significant difference between soil water regimes and pathogen within fungal endophyte treatments at *p* ≤ 0.05 by Tukey’s HSD test. Bars topped by the same uppercase letter indicate no significant difference associated with fungal endophyte at *p* ≤ 0.05 by Tukey’s HSD test.

The chlorophyll content (Figure 2a) and stomatal conductance (Figure 2c) of B+ plants, both infected and uninfected with the fungal endophyte, decreased with a decrease in soil water regime (*p* < 0.05). Pathogen infection decreased the chlorophyll content and transpiration rate (Figure 2d) of E− plants and decreased the net photosynthetic rate (Figure 2b) and stomatal conductance of E+ plants at all soil water regimes (*p* < 0.05). The lowest chlorophyll content and stomatal conductance were obtained in E− plants inoculated with the pathogen and subjected to a 30% soil water regime. The presence of the fungal endophyte significantly increased the photosynthetic parameters. Compared to E− plants, the grass endophyte significantly increased the chlorophyll content, net photosynthetic rate, stomatal conductance, and transpiration rate by 14.3%, 82.9%, 162.5%, and 69.3%, respectively. The E+ B− treatments at 70% soil water had the highest net photosynthetic rate and stomatal conductance (*p* < 0.05; Figure 2b,c). 

### 3.3. Plant Growth

The fungal endophyte enhanced the growth of perennial ryegrass, and the effects were first observed 14 days after seedling transplant into the pots. In this growth period, all E+ seedlings demonstrated growth by 9.0% and 40.0% in shoot height and tiller numbers with the 70% soil water regime, relative to other treatments (Figure S1). 

Fungal endophyte, soil water regime, and the pathogen significantly affected the shoot and root dry weights. Of note, a soil water regime × pathogen interaction was observed in the effects to shoot and root dry weights (Table 1). 

**Figure 3:**
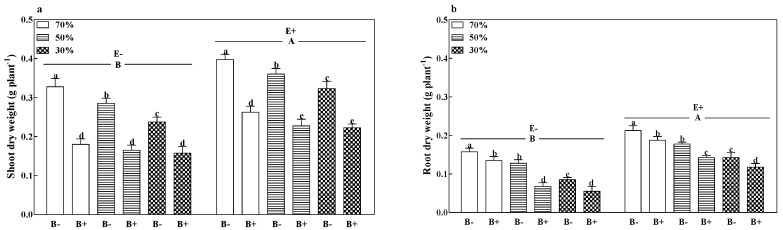
Shoot dry weight (**a**) and root dry weight (**b**) of perennial ryegrass infected with (E+) or without (E−) fungal endophyte and inoculated (B+) or noninoculated (B−) with pathogen at 70%, 50%, and 30% soil water regimes at harvest. Mean ± SEM of four replicates are shown. Bars topped by the same lowercase letter indicate no significant difference between soil water regimes and pathogen within fungal endophyte treatments at *p* ≤ 0.05 by Tukey’s HSD test. Bars topped by the same uppercase letter indicate no significant difference associated with fungal endophyte at *p* ≤ 0.05 by Tukey’s HSD test.

The shoot dry weight (Figure 3a), root dry weight (Figure 3b), and total dry weight (Figure S2) of B− plants and root dry weight of B+ plants decreased with a reduction in soil water regime (*p* < 0.05). Pathogen infection significantly decreased the shoot, root, and total dry weights of both E+ and E− plants at all soil water regimes (*p* < 0.05). Compared to E− plants, the grass endophyte significantly increased the shoot, root, and total dry weights by 32.9%, 56.7%, and 40.4%. The E+ B− treatments at 70% soil water had the highest shoot, root, and total dry weights (Figure 3a,b and Figure S2; *p* < 0.05). 

### 3.4. Phosphorous Contents

**Figure 4:**
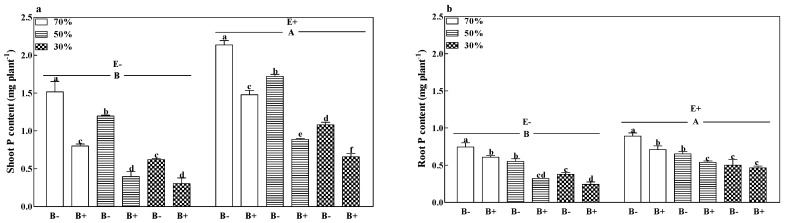
Shoot P content (**a**) and root P content (**b**) of perennial ryegrass infected with (E+) or without (E−) fungal endophyte and inoculated (B+) or noninoculated (B−) with pathogen at 70%, 50%, and 30% soil water regimes at harvest. Mean ± SEM of four replicates are shown. Bars topped by the same lowercase letter indicate no significant difference between soil water regimes and pathogen within fungal endophyte treatments at *p* ≤ 0.05 by Tukey’s HSD test. Bars topped by the same uppercase letter indicate no significant difference associated with fungal endophyte at *p* ≤ 0.05 by Tukey’s HSD test.

The shoot, root, and total P contents of B− plants of both E+ and E− plants decreased with a decrease in soil water regime. The shoot and total P contents of B+ plants infected with the fungal endophyte (Figure 4a,b and Figure S3) were also reduced with a decrease in soil water regime. Generally, pathogen infection significantly decreased the shoot and root P contents of both E+ and E− plants, except for E+ plants at 30% soil water. Compared to E− plants, the fungal endophyte significantly increased the shoot, root, and total P contents by 64.5%, 32.3%, and 53.0%. The E+ B− treatments at 70% soil water had the highest shoot, root, and total P contents (Figure 4a,b and Figure S3; *p* < 0.05).

### 3.5. Enzyme Activity

**Figure 5:**
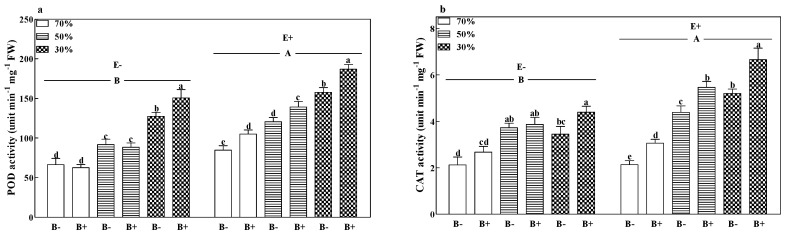
Peroxidase (POD) (**a**) and catalase (CAT) (**b**) enzyme activity of perennial ryegrass infected with (E+) or without (E−) fungal endophyte and inoculated (B+) or noninoculated (B−) with pathogen at 70%, 50%, and 30% soil water regimes at harvest. Mean ± SEM of four replicates are shown. Bars topped by the same lowercase letter indicate no significant difference between soil water regimes and pathogen within fungal endophyte treatments at *p* ≤ 0.05 by Tukey’s HSD test. Bars topped by the same uppercase letter indicate no significant difference associated with fungal endophyte at *p* ≤ 0.05 by Tukey’s HSD test.

The POD activities of all treatments significantly increased with a decrease in the soil water regime (*p* < 0.05). Pathogen infection significantly increased the CAT activities of E+ plants at all soil water regimes. The fungal endophyte significantly increased the POD and CAT activities by 35.3% and 33.06%, respectively (Figure 5a,b; *p* < 0.05).

### 3.6. Proline Content and MDA Concentration

**Figure 6:**
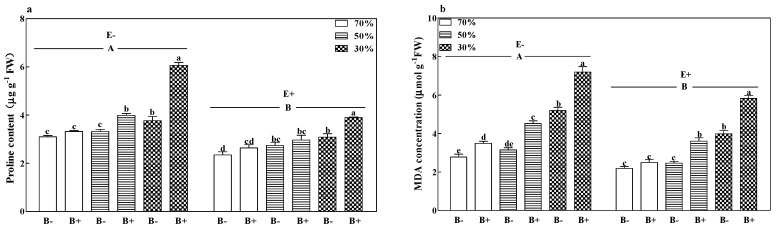
Proline content (**a**), and malonaldehyde (MDA) concentration (**b**) of perennial ryegrass infected with (E+) or without (E−) fungal endophyte and inoculated (B+) or noninoculated (B−) with pathogen at 70%, 50%, and 30% soil water regimes at harvest. Mean ± SEM of four replicates are shown. Bars topped by the same lower case letter indicate no significant difference between soil water regimes and pathogen within fungal endophyte treatments at *p* ≤ 0.05 by Tukey’s HSD test. Bars topped by the same upper case letter indicate no significant difference associated with fungal endophyte at *p* ≤ 0.05 by Tukey’s HSD test.

The malonaldehyde (MDA) concentration of B+ plants (Figure 6a), both E+ and E−, and the proline contents of B+ E− plants (Figure 6b) increased significantly with a decrease in the soil water regime (*p* < 0.05). Pathogen infection increased the MDA concentration of E+ plants and increased the proline content of E− plants at 50% and 30% soil water. Moreover, pathogen infection increased the MDA concentration of E− plants in all soil water regimes and increased the proline contents of E+ plants at 30% soil water (*p* < 0.05). Diseased E− plants had the highest MDA and proline concentrations at 30% soil water. Compared to E− plants, the fungal endophyte in E+ plants significantly decreased MDA concentration and proline content by 21.9% and 25.0%, respectively (Figure 6a,b; *p* < 0.05).

## 4. Discussion

The present study reveals the effects of the fungal endophyte *Epichloë festucae* var. *lolii* on the combined drought and pathogen stresses on perennial ryegrass. The disease incidence of E+ plants increased with decreased soil water content, giving the highest disease incidence at 30% soil water. Compared to the 70% soil water regime, E− plants also gave a higher disease incidence at 50% and 30% soil water. The present study also reveals that the fungal endophyte significantly increased photosynthetic parameters, P uptake, and enzyme activities but decreased the MDA concentration and proline content of perennial ryegrass, even under disease or drought stress. 

In the present study, both E+ and E− demonstrated higher disease incidence at 30% soil water, relative to 70% soil water. Xia et al. [37] also observed the highest disease incidence and index at the lowest water content (15%). These results suggest that drought stress decreases the resistance of plants to the disease. Mitchell et al. [38] attributed this phenomenon to reduced photosynthetic rates due to reductions in stomatal aperture and subsequent lack of enough carbohydrates to sustain normal growth as drought intensifies, thus reducing the ability to resist pathogens. These changes are also related to a reduction in the nutrient uptake capacity of plant roots, inhibition of nutrient transport from plant roots to the shoots, and restriction of transpiration [39,40,41]. The decrease in photosynthetic parameters and nutrient uptake ultimately reduces plant growth [37]. However, Gao et al. [42] found that drought stress to *Apocynum venetum* plants in field conditions enhances their resistance to the rust disease caused by *Melampsora apocyni*. This difference could be associated with differences in environmental conditions, plant species, and pathogen strains. 

Both fungal endophytes and the pathogen obtain carbohydrates from host plants. When the two simultaneously interacted with the host in this study, plants infected with fungal endophytes displayed significantly decreased disease incidence at 70% and 50% soil water. This result indicates that the fungal endophyte shows a competitive relationship with the pathogen at low and moderate soil water deficits. Xia et al. [27] studied the effect of the systemic seed-borne endophyte *Epichloë gansuensis* on powdery mildew disease caused by *Blumeria graminis* on the host grass *A. inebrians* under four soil water conditions (15%, 30%, 45%, and 60%). They found the presence of the fungal endophyte reduced the ability of *B. graminis* to colonize *A. inebrians* at all tested soil water conditions. This finding may be because the fungal endophyte could kill or inhibit pathogen growth, and the mechanisms could involve competition for nutrients and space, the production of metabolites with antifungal activity, or induction of the plant immune system [8]. Fungal endophytes enhance plant resistance to pathogens by inhibiting sporulation and spore germination [43,44] or preventing pathogen invasion, colonization, and development [45]. 

In this study, the *Bipolaris sorokiniana* infection significantly decreased the shoot, root, and total dry weights of ryegrass at all soil water regimes. These results are similar to our previous studies [19,46], which showed that *B. sorokiniana* significantly decreases the total dry weight of ryegrass at normal soil water conditions. This result is primarily related to the lower photosynthesis of pathogen-infected plants that is observed in this study. The lower photosynthetic rates imply that the plant is more likely to grow slower [47], which reduces the plant yields further [48,49,50]. Moreover, the reduction of nutrient uptake also results in lower shoot and root dry weights because P is essential for plant development and growth, making up about 0.2% of the dry weight of plants [51]. 

Similar to our previous finding [19], the present study showed that E+ plants had higher shoot, root, and total dry weights. Xia et al. [27] also reported that E+ plants show significantly higher fresh and dry weights than E− plants with increasing soil water contents, regardless of *B. graminis* infection. This result further indicates that the grass endophyte improves plant growth. Zhang et al. [52] determined the effects of the fungal endophyte *E. gansuensis* on drunken horse grass over a 4-year period under field conditions; they found that endophyte infection is beneficial to growth, seed yield, and plant survival of this grass species. Malinowski and Belesky [53] found that when no P was supplied, fungal endophyte infection reduced shoot dry matter by 20%, while the relative growth rate of E+ plants was 16% greater than that of E− plants when P was supplied as phosphate rock at 25 mg kg-1. Guo et al. [46] also reported that fungal endophytes decrease the biomass of ryegrass under limited soil nutrients. These results suggest that the role of fungal endophytes is primarily associated with environmental conditions. 

The fungal endophyte mitigated the adverse effects of the pathogen by increasing the net photosynthetic rate and stomatal conductance of ryegrass in the present study. Amalric et al. [54] also found that E+ ryegrass has higher values for photosynthetic parameters than E− plants, and infection by fungal endophytes increases the tiller number of ryegrass by 30%. However, Pinto et al. [55] reported that E+ maize plants showed a 50% reduction in total chlorophyll content and severely reduced photosynthetic capacity. These result disparities could be attributed to differences in plant species, endophyte species, and environmental conditions. Moreover, the plants in this study that were infected with the fungal endophyte also had higher P contents. This finding indicates that the fungal endophyte not only offers advantages in nutrient acquisition but also changes plant photosynthesis in response to disease and drought stresses.

Stressed plants increase the production of reactive oxygen species (ROS), increasing lipid peroxidation [56]. The higher accumulation of MDA is related to a high degree of lipid peroxidation in plant cells, which results in severe damage to the cell membrane [57]. In this study, the combination of pathogen infection and severe drought (30% soil water regime) increased the plant’s MDA concentration and proline contents. This result indicates that the combined pathogen and drought stresses inflicted more severe damage to plants. However, the POD activity of diseased perennial ryegrass was stimulated and maintained at high levels in both E+ and E− plants exposed to severe drought (30% soil water). This finding is mainly because the enhanced activity of antioxidant enzymes such as CAT and POD can remove ROS to prevent or reduce cell injury [58]. Various studies on different fungal endophytes have supported that the presence of endophytes increases the host’s stress tolerance capacity due to increased antioxidant activity, which increases ROS scavenging rates and lowers ROS accumulation in E+ host tissues [59,60,61]. Zhang and Nan [61] found that the fungal endophyte increases seedling growth and biomass accumulation in *Elymus dahuricus* under drought stress. The increased growth in response to drought resulted, at least in part, from higher antioxidant activity. They also found a positive effect of endophyte colonization on proline concentrations under low water conditions. In this study, E+ plants demonstrated higher activities of antioxidant enzymes than E− plants under stress conditions, which concurs with Ma et al. [20] and Li et al. [19]. This result could suggest that perennial ryegrass infected with the fungal endophyte developed mechanisms to reduce oxidative damage following plant exposure to biotic and abiotic stresses [62].

## 5. Conclusions

The current study assessed the effects of an *Epichloë* fungal endophyte on its host grass when exposed to combined pathogen and drought stresses. The fungal endophyte increased P uptake, plant growth, and photosynthetic parameters but decreased malondialdehyde concentration, proline content, and disease incidence of perennial ryegrass. E+ plants had the lowest disease incidence at 70% soil water. The study demonstrated that the fungal endophyte *E. festucae* var. *lolii* is beneficial for plant growth and stress tolerance in perennial ryegrass exposed to the combined stresses of drought and *B. sorokiniana*. These results are helpful to elucidate the physiological mechanisms of the plant–*Epichloë* symbiosis, provide a basis for conducting similar evaluations under field conditions, and can potentially be applied to other plant–*Epichloë* symbiosis systems in the world.

## Figures and Tables

**Table 1:** ANOVA result for effects of fungal endophyte (E), *B. Sorokiniana* (B), and soil water regime (W), and their interactions on the listed variables.

Treatments		Grass Endophyte (E)	Soil Water Content (W)	*B. Sorokiniana* (B)	Interactions			
					ExW	ExB	WxB	ExWxB
df		1	1	2	3	5	5	11
Disease incidence	F	155.033	152.409	27161.57	24.179	155.033	152.409	24.179
	*P*	<0.001	<0.001	<0.001	<0.001	<0.001	<0.001	<0.001
Chlorophyll content	F	52.782	259.563	137.334	3.515	11.45	27.835	0.701
	*P*	<0.001	<0.001	<0.001	0.040	0.002	<0.001	0.503
Net photosynthetic rate	F	1018.041	244.494	319.359	28.523	2.636	13.831	21.097
	*P*	<0.001	<0.001	<0.001	<0.001	0.118	<0.001	<0.001
Stomatal conductance	F	1705.375	593.57	216.573	208.985	11.202	9.737	3.626
	*P*	<0.001	<0.001	<0.001	<0.001	0.003	0.001	0.042
Transpiration rate	F	740.504	624.127	2351.147	76.481	32.306	262.622	55.414
	*P*	<0.001	<0.001	<0.001	<0.001	<0.001	<0.001	<0.001
Shoot DW	F	296.05	57.346	777.243	0.811	0.603	12.779	1.779
	*P*	<0.001	<0.001	<0.001	0.453	0.443	<0.001	0.183
Root DW	F	483.049	254.449	153.646	1.712	3.917	8.824	2.867
	*P*	0.003	<0.001	<0.001	0.195	0.055	0.001	0.070
Total DW	F	908.904	291.632	1208.389	0.111	0.196	17.12	1.056
	*P*	<0.001	<0.001	<0.001	0.895	0.661	<0.001	0.359
Shoot P content	F	734.088	604.166	1058.215	13.315	0.523	47.757	1.511
	*P*	<0.001	<0.001	<0.001	<0.001	0.477	<0.001	0.241
Root P content	F	135.529	232.609	111.113	1.168	4.661	4.215	3.677
	*P*	<0.001	<0.001	<0.001	0.328	0.041	0.027	0.040
Total P content	F	720.775	703.723	922.228	5.065	0.38	40.04	0.355
	*P*	<0.001	<0.001	<0.001	0.015	0.544	<0.001	0.705
POD	F	260.567	427.909	43.127	1.747	16.454	8.105	1.755
	*P*	<0.001	<0.001	<0.001	0.196	<0.001	0.002	0.194
CAT	F	138.935	241.141	80.877	30.678	10.288	3.639	0.817
	*P*	<0.001	<0.001	<0.001	<0.001	0.004	0.042	0.454
MDA	F	330.853	1023.702	542.854	9.4	6.157	59.414	0.402
	*P*	<0.001	<0.001	<0.001	0.001	0.020	<0.001	0.673
Proline	F	589.56	392.786	346.325	30.919	58.571	99.729	32.424
	*P*	<0.001	<0.001	<0.001	<0.001	<0.001	<0.001	<0.001

## Supplementary Materials

The following are available online at https://www.mdpi.com/2076-2607/8/12/1917/s1. Figure S1: Shoot height (**a**) and tiller number (**b**) of perennial ryegrass infected with (E+) or without (E−) fungal endophyte at 70%, 50%, and 30% soil water regimes before inoculated by *B. sorokiniana*. Figure S2: Total dry weight of perennial ryegrass infected with (E+) or without (E−) fungal endophyte and inoculated (B+) or noninoculated (B−) with pathogen at 70%, 50%, and 30% soil water regimes at harvest. Figure S3: Total P content of perennial ryegrass infected with (E+) or without (E−) fungal endophyte and inoculated (B+) or noninoculated (B−) with pathogen at 70%, 50%, and 30% soil water regimes at harvest.
