# Supplementary material for: Effects of the Fungal Endophyte Epichloë festucae var. lolii on Growth and Physiological Responses of Perennial Ryegrass cv. Fairway to Combined Drought and Pathogen Stresses
Source: Microorganisms. 2020 Dec 2;8(12):1917. doi: 10.3390/microorganisms8121917 (PMC7760724; doi:10.3390/microorganisms8121917)
Supplement: Supplementary file 1 [file microorganisms-08-01917-s001.pdf]

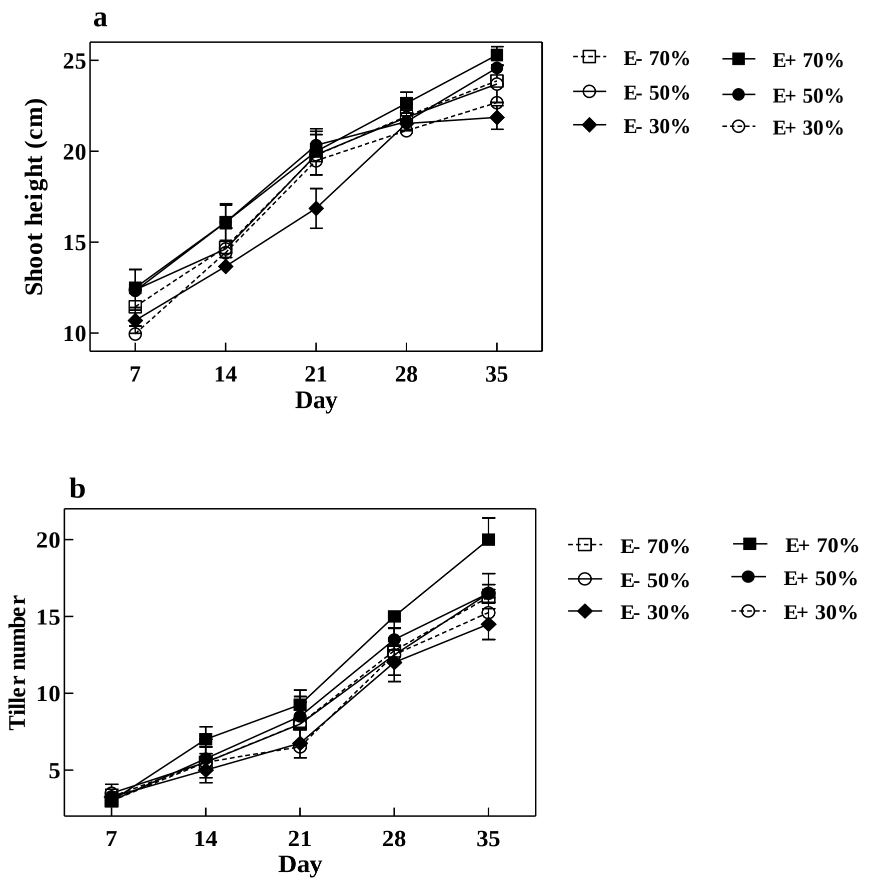

Supplementary Figure S1. Shoot height (a) and tiller number (b) of perennial ryegrass infected with (E+) or without (E-) fungal endophyte at 70%, 50%, and 30% soil water regime before inoculated by *B. sorokiniana*. Mean ± SEM of four replicates.

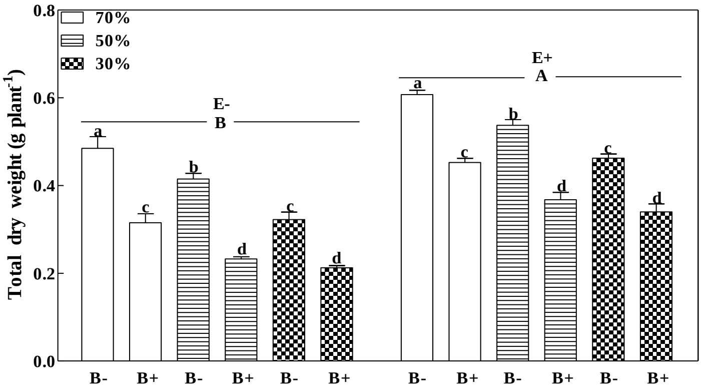

Supplementary Figure S2. Total dry weight of perennial ryegrass infected with (E+) or without (E-) fungal endophyte and inoculated (B+) or non-inoculated (B-) with pathogen at 70%, 50%, and 30% soil water regime at harvest. Mean ± SEM of four replicates are shown. Bars topped by the same lowercase letter indicate no significant difference between soil water regimes and pathogen within fungal endophyte treatments at $P \leq 0.05$ by Tukey's HSD test. Bars topped by the same uppercase letter indicate no significant difference associated with fungal endophyte at $P \leq 0.05$ by Tukey's HSD test.

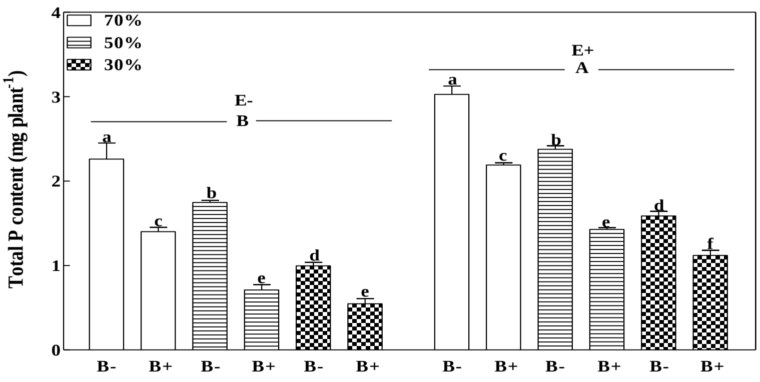

Supplementary Figure S3. Total P content of perennial ryegrass infected with (E+) or without (E-) fungal endophyte and inoculated (B+) or non-inoculated (B-) with pathogen at 70%, 50%, and 30% soil water regime at harvest. Mean ± SEM of four replicates are shown. Bars topped by the same lowercase letter indicate no significant difference between soil water regimes and pathogen within fungal endophyte treatments at $P \leq 0.05$ by Tukey's HSD test. Bars topped by the same uppercase letter indicate no significant difference associated with fungal endophyte at $P \leq 0.05$ by Tukey's HSD test.
